# Pioneering Physician: Dr. Elizabeth Blackwell and the Path to Medical History

**DOI:** 10.7759/cureus.68713

**Published:** 2024-09-05

**Authors:** Janhvi Thakur, Sonali G Choudhari, Abhay Gaidhane

**Affiliations:** 1 Department of Community Medicine, Jawaharlal Nehru Medical College, Datta Meghe Institute of Higher Education and Research, Wardha, IND

**Keywords:** gender equality, health reform, historical vignette, medical education, women physician

## Abstract

Elizabeth Blackwell was a remarkable and courageous woman who made significant contributions to medicine, education, and the rights of women. Blackwell achieved significant milestones in the medical field by being the first female physician in America and the first woman to be listed on the United Kingdom medical register. Despite facing challenges, discrimination, and barriers, she founded the London School of Medicine and the New York Infirmary for Women and Children to provide better education and health benefits for women and children. She lived during a time when women were expected to stay in the home; yet, she went on to become a doctor and professor.

## Introduction and background

**Figure 1:**
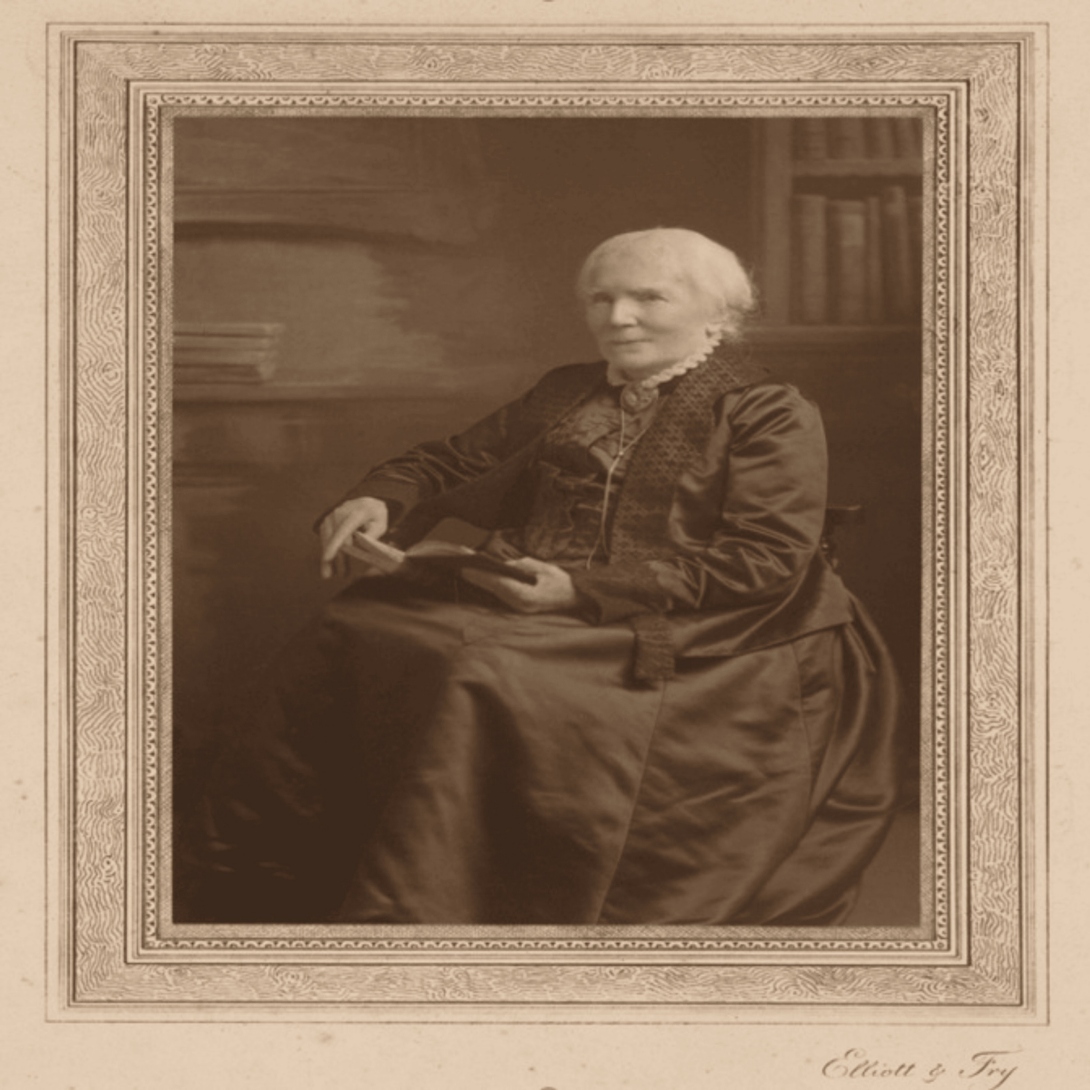
Dr. Elizabeth Blackwell (1821-1910) Artist: Elliott and Fry Source: National Portrait Gallery, Smithsonian Institution [2]. This image is under Creative Commons Zero.

This article aims to highlight the remarkable achievements of Dr. Elizabeth Blackwell, a trailblazing figure in the medical field and the most powerful American woman in Bristol. She made history by becoming the first American woman to graduate with a medical degree. In 1859, Blackwell achieved another milestone by being the first woman to be officially registered with the medical register of the British General Medical Council. Blackwell was a pioneer who launched several leading-edge health programs, played a key role in numerous reform initiatives, and dedicated her life to improving healthcare. She significantly advanced the education and acquisition of female physicians in the United States [1]. The portrait of Dr. Elizabeth is shown in Figure 1 [2].

## Review

Early life and education

Elizabeth Blackwell was born in Bristol, England, on February 3, 1821. In 1832, when Dr. Blackwell was just 11, her father's business failed, and her family relocated to the United States. Following a period spent in New York, they moved to Jersey City, New Jersey, in 1835, and later settled in Cincinnati, Ohio, in 1838 [3,4]. There was a strong sense of change, rebellion, and progressive political thought throughout the close-knit family. For instance, they supported equal and free education for men and women, which was a new idea at the time. She was a school teacher, but she also studied medicine on her own under Dr. Samuel Dickson [5]. According to Dr. Blackwell, it was not the first time a woman practised medicine or gained recognition as a medical professional. Since ancient times, women have been practising medicine both publicly and privately [5]. Dr. Blackwell was a dedicated and passionate supporter of women in medicine until she died in 1910, prioritizing women's rights campaigns and establishing medical schools for female students in the United States and the United Kingdom. Dr. Blackwell founded the New York Infirmary for Women and Children in 1858, inspired by the difficulties she encountered as a minority in the medical field. The institution aimed to train female students in medicine and employ female physicians, in addition to providing healthcare for people in need [6].

The journey

Even for men, choosing to become a physician was not an easy decision during the 1840s. At the time, human health was generally poor. A concurrent rise in disease transmission was caused by the development of cities next to polluted water sources, a lack of facilities for the disposal of waste, and a large vermin population. Without a clear cause, diseases like cholera, typhoid fever, tuberculosis, and influenza spread quickly. The germ hypothesis of disease had not yet been proposed by Louis Pasteur [7]. For Blackwell, the driving force was to prove that women could study and practice medicine with the same qualifications as men, and the recognition that this would bring [7]. She wrote in her memoirs that “the idea of winning a doctor’s degree gradually assumed the aspect of a great moral struggle, and the moral fight possessed immense attraction for me” [8].

To ascertain someone or an organization willing to give an opportunity to teach a woman in medical science, Blackwell began calling physicians and medical schools. She wrote to several family friends who were physicians, and every one of them responded in the same way. In her autobiography, she recalled, "They all shared a similar view that, although the idea was valuable, it could not be carried out; that a woman could not receive such an education; that the required education was lengthy and costly; that there were many barriers in the way of such a course; and that, in short, the idea, though valuable, could not be executed." A medical degree was particularly challenging because of the unstable financial situation of the Blackwell family. Blackwell was supported by the profits from her father's sugar plant while growing up in England and then New York. However, her father passed away in 1838, not long after the family had moved to Cincinnati, leaving his wife and nine children with only $20 in his bank account [7]. Elizabeth worked as a teacher at Asheville Female Seminary to support her family because it was one of the only employment options open to women in the antebellum period [7].

While Elizabeth was in her mid-20s, her close companion passed away following a long ailment. Her ill friend expressed that she would not have suffered as much if she had been allowed to see a female physician. This was the tipping point when Elizabeth decided to devote her career to studying medicine and assisting women in accessing high-quality healthcare. She spent a few years studying medicine in private before applying to Geneva Medical College. The college took the opinion of male students into account as well, to decide the acceptance of Elizabeth's application. Elizabeth enrolled in medical school when the students voted "yes," and she made history by becoming the first female to be registered in the British General Medical Council [9].

Talk of the town

Blackwell faced racial discrimination during her college days. She was forced to sit in a separate row by her professors and was often prevented from attending labs, while the community frequently humiliated her as she pursued her education and career goals against the prevailing customs and culture. In 1849, Blackwell finally proved herself to her teachers and companions by ranking first in the class. Although doctors had advised her to restrict herself only to midwifery or nursing training, she nonetheless pursued both fields in London and Paris hospitals. Faced with male doctors who consistently failed to practice better hygiene by not washing their hands while treating patients, she became more interested in preventive care and personal hygiene, as these are crucial aspects of maintaining overall health and well-being. Despite being the only female medical student at the school, she was the talk of the town, and other women disapproved of her for challenging gender norms [3,6].

Blackwell’s academic success

Elizabeth conducted an observational study at Blockley Almshouse in Philadelphia during the spring and summer semester break. Although she obtained approval from the regulatory agency to observe patients and medical personnel, she faced resistance from the resident physicians, who declined to collaborate with her. Blockley Almshouse served as a treatment centre for the city's most vulnerable population, including impoverished individuals and Irish immigrants suffering from typhus, also known as "ship fever." Elizabeth's thesis, based on her observations and research, was highly acclaimed and selected for publication in the Buffalo Medical Journal, a prestigious scientific platform [10].

Challenges and barriers

In April, after obtaining her naturalization as a citizen of the United States, Blackwell moved to England in May to pursue additional training, and in June, she enrolled in La Maternité's midwifery program in Paris. She contracted an infectious condition of the eyes while attending to a newborn who had an eye infection caused by bacteria - most likely gonorrhoea acquired from the baby's mother during delivery - which rendered one of her eyes blind and forced her to give up on her aim of being a surgeon. She moved back to England in October 1850 and began working for Dr. (later Sir) James Paget at St. Bartholomew's Hospital. She returned to New York in 1851, but this time, she was turned down for jobs in the city's hospitals and pharmacies and was unable to even afford private consulting rooms. Her private practice took a long time to develop, so she authored a lecture series that was eventually published in 1852 as *The Laws of Life, With Special Reference to the Physical Education of Girls* [1,11].

Key achievements

First Women-Managed Infirmary in the United States

Dr. Blackwell had the dispensary extended into the New York Infirmary for Women and Children. In 1857, she transitioned from operating a part-time dispensary with limited services to establishing the New York Infirmary for impoverished women and children, a comprehensive healthcare facility offering round-the-clock care for medical and surgical patients. This innovative healthcare institution had a dual mission: first, to provide accessible healthcare services to underserved populations, and second, to serve as a professional development hub for female physicians, medical students, and nursing scholars, addressing the gender gap in medical education and practice. By providing a dedicated training environment, the infirmary aimed to enhance the skills and expertise of women in healthcare, promoting gender equality in the medical field [11,12]. After making several trips throughout Europe, Dr. Blackwell developed a growing interest in social reform movements that supported Christian socialism, women's rights, family planning, hygiene, eugenics, medical education, and sexual purity. Throughout the 1860s and 1870s, she made several trips back to London, where she assisted in the establishment of the London School of Medicine for Women in 1874-1875 [11,13].

Providing Women With a Safe Environment to Study

Elizabeth advocated for women, arguing that they should study medicine in the same recognized institutions as men. She recognized a gender-based admission bias in medical colleges, which hindered female professionals' education. This led her to set up a women's medical college, addressing systemic inequality. Her initiative promoted gender inclusivity in medical education. When the Woman's Medical College of the New York Infirmary first opened its doors in 1868, it had 15 students and 9 teaching members, including Emily, Elizabeth's younger sister, who was a professor of obstetrics and women's disorders, and Elizabeth, who was a professor of hygiene. The college was managed by Emily after Elizabeth moved to England the year after it opened. Having always intended to pursue her profession in England, she left New York in 1869 and spent the next 40 years there [12].

Emphasized Women's Health and Hygiene

Elizabeth Blackwell emphasized the importance of cleanliness and public health throughout her career as a means of preventing illness and enhancing overall well-being. She was an outspoken supporter of hygiene, sanitation, and preventive medicine [13].

Establishment of the National Health Society

In 1871, Elizabeth contributed to the establishment of the National Health Society, a prominent organization dedicated to promoting public health. She also successfully developed a thriving private medical practice, demonstrating her clinical expertise. Furthermore, in 1875, she was appointed as a professor of gynaecology at the London School of Medicine for Women, a prestigious academic institution. This appointment recognized her authority in the field of gynaecology and enabled her to share her knowledge and experience with future generations of female medical professionals, contributing to the advancement of women in medicine and the development of obstetric and gynaecologic care [1].

It is remarkable that, at the time of Dr. Blackwell's death in 1910, women made up about 6% of American physicians. By 2021, women accounted for 35% of practising physicians and more than half of medical students in the United States [7].

A Deft Musician

Before pursuing medicine, Blackwell showcased her musical talent by teaching at an academy in North Carolina, using her musical skills to fund her education. This lesser-known aspect of her life highlights her multifaceted abilities, revealing a talented musician behind the renowned physician [13].

## Conclusions

Elizabeth Blackwell’s pioneering spirit, courage, and dedication to her craft have left an indelible mark on the history of medicine. Despite facing numerous obstacles, she made a great contribution to advocating for women’s admission to medical school, fighting for the rights of women and gender equality, as well as emphasizing public health and hygiene. She was the first woman who inspired other women to pursue medical careers. Her contributions toward better health will forever be remembered and celebrated.
